# Impact of customized add-on nighttime bracing in full-time brace treatment of adolescent idiopathic scoliosis

**DOI:** 10.1371/journal.pone.0278421

**Published:** 2023-01-26

**Authors:** Henriette Bretschneider, Peter Bernstein, Alexander C. Disch, Jens Seifert

**Affiliations:** 1 University Center of Orthopaedic, Trauma & Plastic Surgery (OUPC), University Hospital Carl Gustav Carus of Technische Universität Dresden, Dresden, Germany; 2 University Comprehensive Spine Center (UCSC), University Hospital Carl Gustav Carus of Technische Universität Dresden, Dresden, Germany; 3 Department of Spine Surgery, AKG Klinik Hohwald GmbH, Neustadt in Sachsen, Germany; Universitair Kinderziekenhuis Koningin Fabiola: Hopital Universitaire des Enfants Reine Fabiola, BELGIUM

## Abstract

**Study design:**

Retrospective cohort study.

**Objective:**

Bracing is an accepted standard therapy for idiopathic scoliosis at Cobb angle ranges between 25° and 40°. However, it is unclear, if a specifically tailored regimen of daytime and nighttime braces (= double brace) yields superior results compared to the standard treatment (single brace for day and night).

**Methods:**

One-hundred-fifteen patients with adolescent idiopathic scoliosis (AIS) were assessed before initiation of bracing treatment and at the final follow-up 2 years after deposition of the brace. They were divided into two groups: double-brace group (n = 66, 4 male, 62 female, age 13.1 ± 1.9 (mean ± SD), primary curvature thoracic n = 35, lumbar n = 31) and single-brace group (n = 49, 8 male, 41 female, age 14.1 ± 1.9, primary curvature thoracic n = 18, lumbar n = 31). Each patient underwent clinical and radiological examinations and Cobb angles were measured.

**Results:**

Both therapy regimens succeeded to either stop progression or improve scoliosis in over 85% of cases. The nighttime brace showed a significantly higher primary correction than the daytime brace. Nevertheless, there was no significant difference in treatment success in the 2-year follow-up (p = 0.58).

**Conclusion:**

It seems to be sufficient to treat idiopathic scoliosis with one well-tailored brace for day- and nighttime.

## 1. Introduction

**Fig 1:**
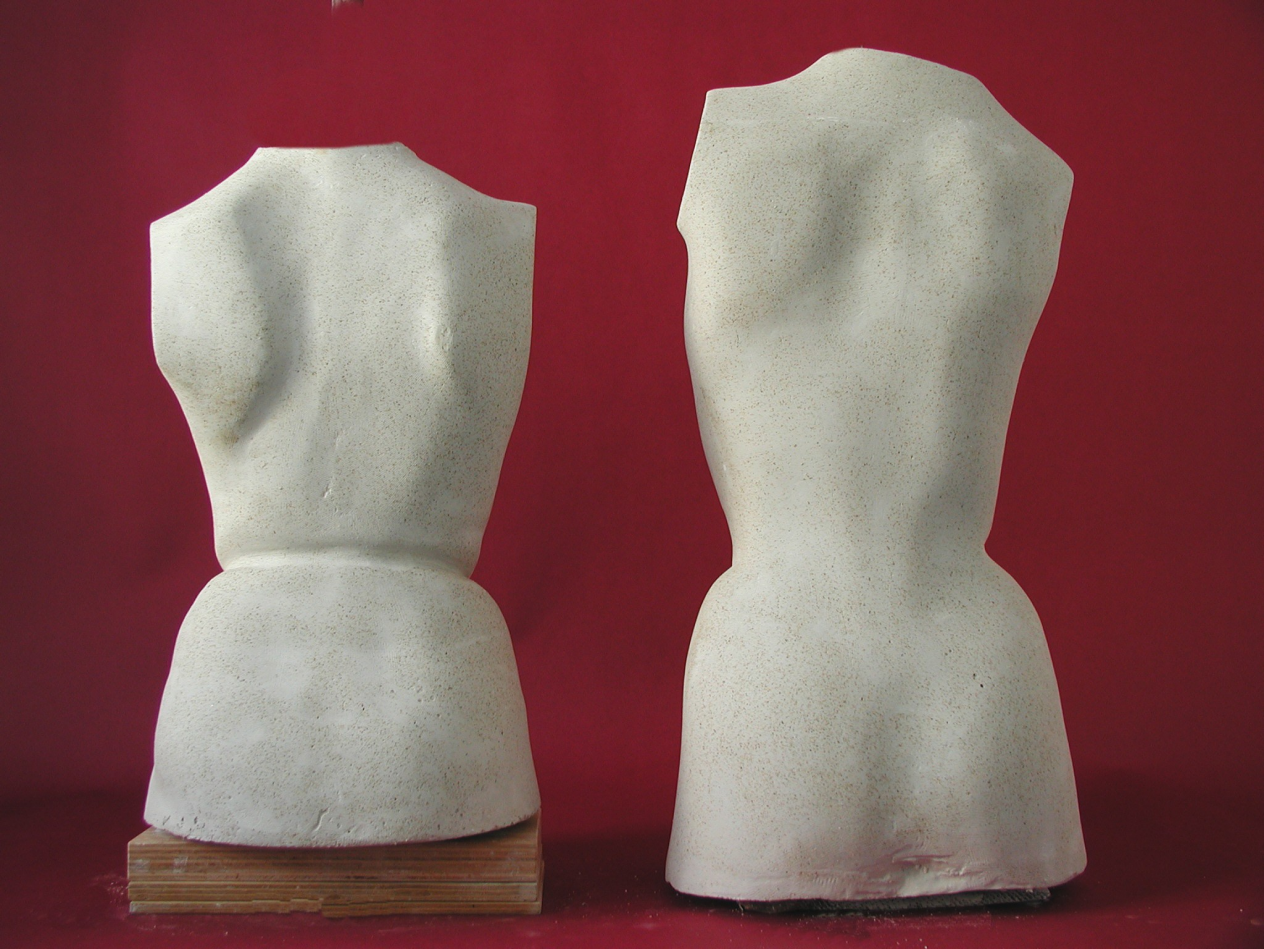
Plaster model of a patient with AIS—standing position (left) and lying position (right)–thus demonstrating the gravity dependent change of trunk shape.

**Fig 2:**
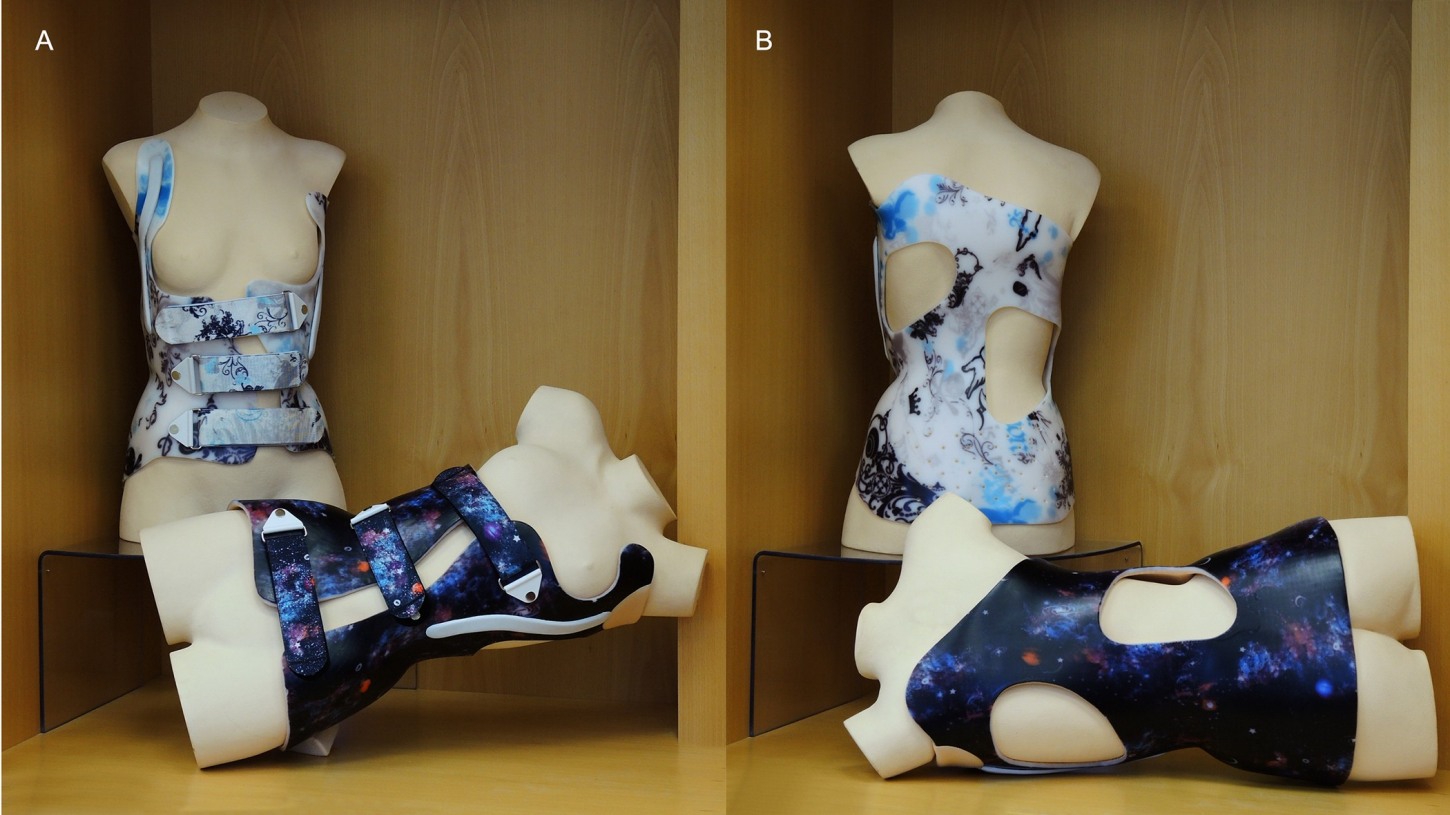
Exemplary presentation of a the Chêneau brace (upright position, to be worn at daytime) and the Charleston approach (horizontal position, to be worn at night) in front (A) and rear (B) view.

Bracing is acknowledged as standard therapy for idiopathic scoliosis at Cobb angle ranges between 25° and 40° [1]. Braces aim at preventing spinal curve deterioration beyond the point of 45° or surgery in order to preserve long-term life quality. They should be applied in growing children and adolescents only. The Scoliosis Research Society has established widely accepted criteria to define whether a child is eligible for bracing and how treatment results should be evaluated. Those include patient parameters (skeletal immaturity, Cobb angle) and study design patterns (follow-up at 2 years, curve progression defined as >5°deterioration and number of failures (curve > 45° +/- surgery) [2]. As the amount of primary correction and brace acceptance had been identified as key factors for treatment success, efforts were undertaken to maximize patient comfort while maintaining overall good correction [3]. Although full-time bracing has been shown to have detrimental effects on the quality of life, it has been acknowledged by current standards to be the most effective therapeutic approach in the aforementioned Cobb angle range [1, 4–6]. Rigid bracing has proven to achieve superior results over elastic designs [7]. The in-brace correction is a good predictor for final results [3, 8]. The Chêneau brace has proven to be effective over decades since its development in 1978 [9–11]. It works through multipoint pressure zones (e.g. rib hump) and contralateral void spaces to allow for effective derotation. Despite overall good results in most studies, the Chêneau design is challenged in certain cases: impaired quality of life [4], inferior correction of thoracic curves compared with thoracolumbar and lumbar curves [12] and inferior results in double major curve types [13]. Additionally, trunk shape is subject to elongation when changing patient position to the horizontal, which results in brace malfitting and subsequent loss of correction (Fig 1). In order to overcome those limitations, efforts were undertaken to maximize correction, especially in the main thoracic curve while preserving patient compliance. The Charleston bending brace was designed to be worn at night and exerts its compressive forces on the curve convexity through a bending moment, achieved by the elevation of the contralateral shoulder [14]. Although some results seemed to be promising, especially in terms of patient’s acceptance, nighttime bracing alone could not be advised over full-time bracing as only mild curve types achieved sufficient results [15, 16]. From the aforementioned data it becomes clear that there cannot be an ideal brace type to achieve superior treatment results. To achieve a synergistic effect we combined the Chêneau (to be worn duing daytime) and the Charleston approach (to be worn at night) as a double-brace treatment (Fig 2).

The aim of the present study was to compare a double-brace full-day treatment with a single brace full-day treatment in patients with AIS. We analyzed initial Cobb angle, primary correction (primary outcome measure) and Cobb angle at follow-up (2 years after completion of brace weaning, secondary outcome measure) and necessity for surgery.

## 2. Materials and methods

This study was designed as a non-interventional, retrospective cohort study.

Patients were included between 1997 and 2018. The study was approved according to the local institutional review board (IRB) (#EK 27012018). This project received an exemption from the IRB for informed consent, therefore, informed consent was not obtained.

### 2.1. Patient cohort

**Fig 3:**
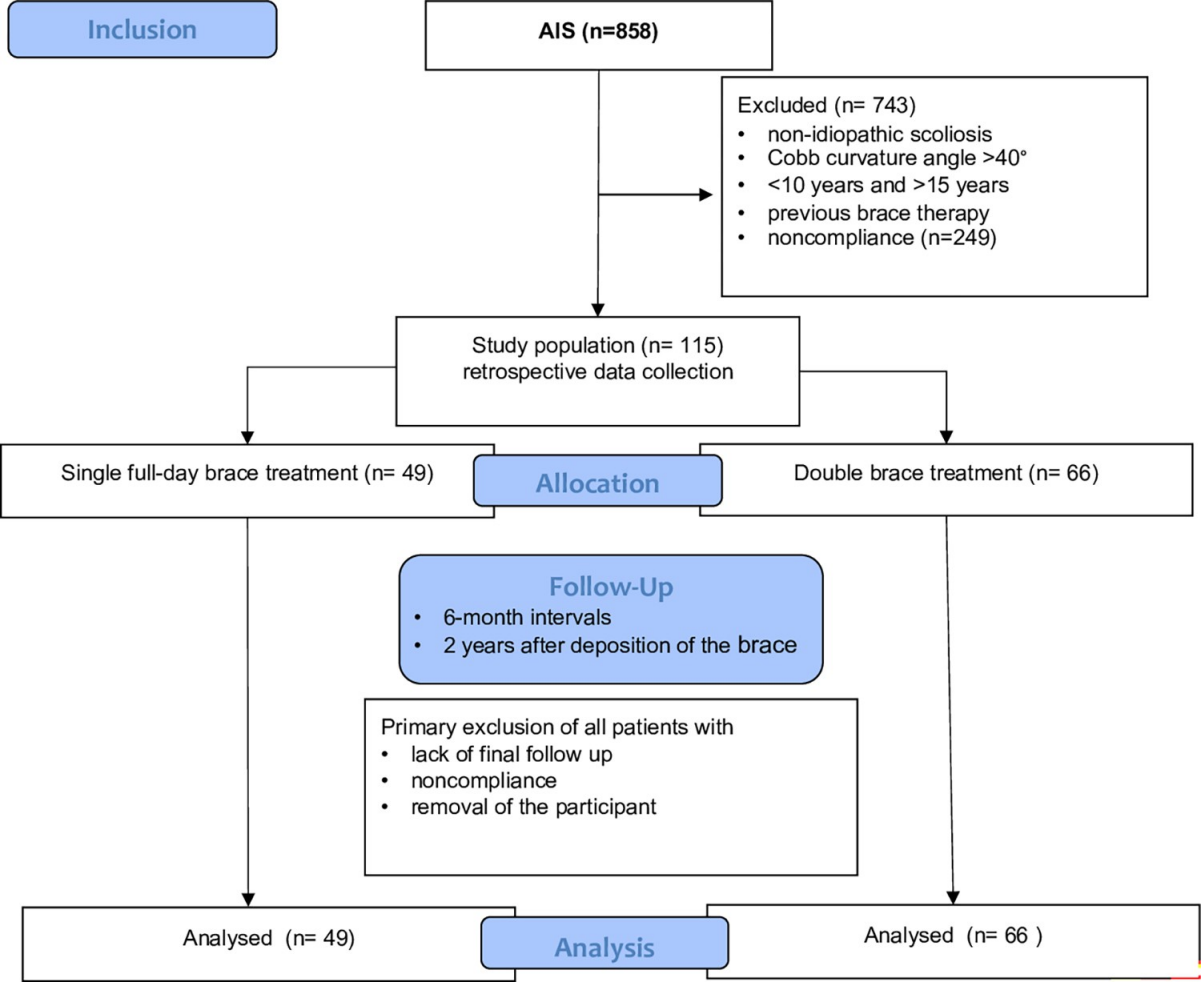
Flowchart of the study (CONSORT document).

Patients were managed as recommended by SOSORT Consensus statement [17]. Inclusion criteria were according to the Scoliosis Research Society criteria an age between 10 and 15 years (y), Risser’s sign of 0–2, Cobb curvature angle of 25–40°, no previous treatment, AIS and compliance (at least 23 h wearing time) [18]. Compliance was documented and questioned based on medical history, adherence to appointments, wear marks on the skin and on the brace. Patients with non-idiopathic scoliosis (neurogenic, secondary or congenital genesis), non-compliance, Cobb curvature angle >40°, <10 years and >15 years for initial treatment were excluded (Fig 3). Patients who were initially treated with a night-time brace only were also excluded. Initial diagnostics included a clinical examination, standardized X-ray of the entire spine in standing position.

Patients for whom cost coverage of double-brace therapy was denied by the health insurance company received a single full-day and nighttime Chêneau type brace treatment. All other patients received a Chêneau brace for daytime and a Charleston type nighttime bending brace. All patients also received prescriptions for once or twice weekly guided physiotherapy. Patients who were prescribed the brace were instructed to wear it for 23 hours/day. Patients were seen at 6-month intervals, at which times we collected radiographic, clinical, orthotic, and self-reported data. Wearing traces were checked during the follow-up visits. Patients who reported a daily wearing time of less than 23 hours were excluded from the study. Brace weaning was started once the patient had reached Risser stage 4 and did not show any further growth according to length measurements. Patient relevant outcome was assessed by clinical examination and standardized X-ray of the entire spine 2 years after deposition of the brace. Traditional COBB measurements on digital whole spine standing radiographs were done by trained orthopedic surgeons.

### 2.2. Bracing

Most braces (93%) were made by a single specialized orthopedic technician. They were designed to provide best possible fit, correction effect and cosmetics. The manufacturing was done in an individual process via plaster casts. A Chêneau-type brace was made to be worn as a full-time brace or during daytime only (double-brace group) [9], whereas the nighttime brace was produced according to the Charleston approach [14]. The braces were fitted with marked pads and windows in addition to bending forces [16]. Brace fitting was checked and if necessary, adjusted at 6-month intervals within the context of the outpatient presentation by a specialized orthopedic technician.

### 2.3. Statistical analysis

Successful treatment was defined as ≤ 5°curve progression. Curve progression >5° and curve > 45° +/- surgery was defined as treatment failure. Numerical data were statistically analyzed using GraphPad Prism 5.04 software (San Diego, CA, USA). Statistical analysis was performed on quantified data using 1-way ANOVA and Tukey´s multiple comparisons test for statistical analysis between the groups. Mean values of each parameter were also compared at brace initiation and at the final follow-up by using Fisher‘s Exact Test 2 –tailed. A p-value ≤ 0.05 was considered significant.

## 3. Results

### 3.1. Patient cohort

We analyzed the brace-treatment course of 115 non-randomized compliant individuals with AIS in a retrospective manner. They were divided into two groups: double-brace group (n = 66, 4 male, 62 female) and single-brace group (n = 49, 8 male, 41 female). All patients were between 10 and 15 years old (double-brace age 13.1 ± 1.9, single-brace age 14.1 ± 1.9 (mean ± SD) t-Test p = 0.03) at the start of the brace treatment and had Risser stage <3 and Cobb curvature angle of 25–40°. Initial curve magnitude was 27° ± 7° (T-Spine) and 25° ± 7° (L-Spine) in the double-brace group and in the single- brace group 26° ± 6° (T-Spine) and 27° ± 7° (L-Spine) (mean ± SD) (T-Spine p = 0.70, L-Spine p = 0.08). Thereby the primary curvature was thoracic in 35 cases and lumbar in 31 cases (double-brace group) respectively thoracic in 18 cases and lumbar in 31 cases (single-brace group) (Fisher‘s Exact Test 2 –tailed, difference in primary curvature p = 0.09).

### 3.2. Primary correction

**Fig 4:**
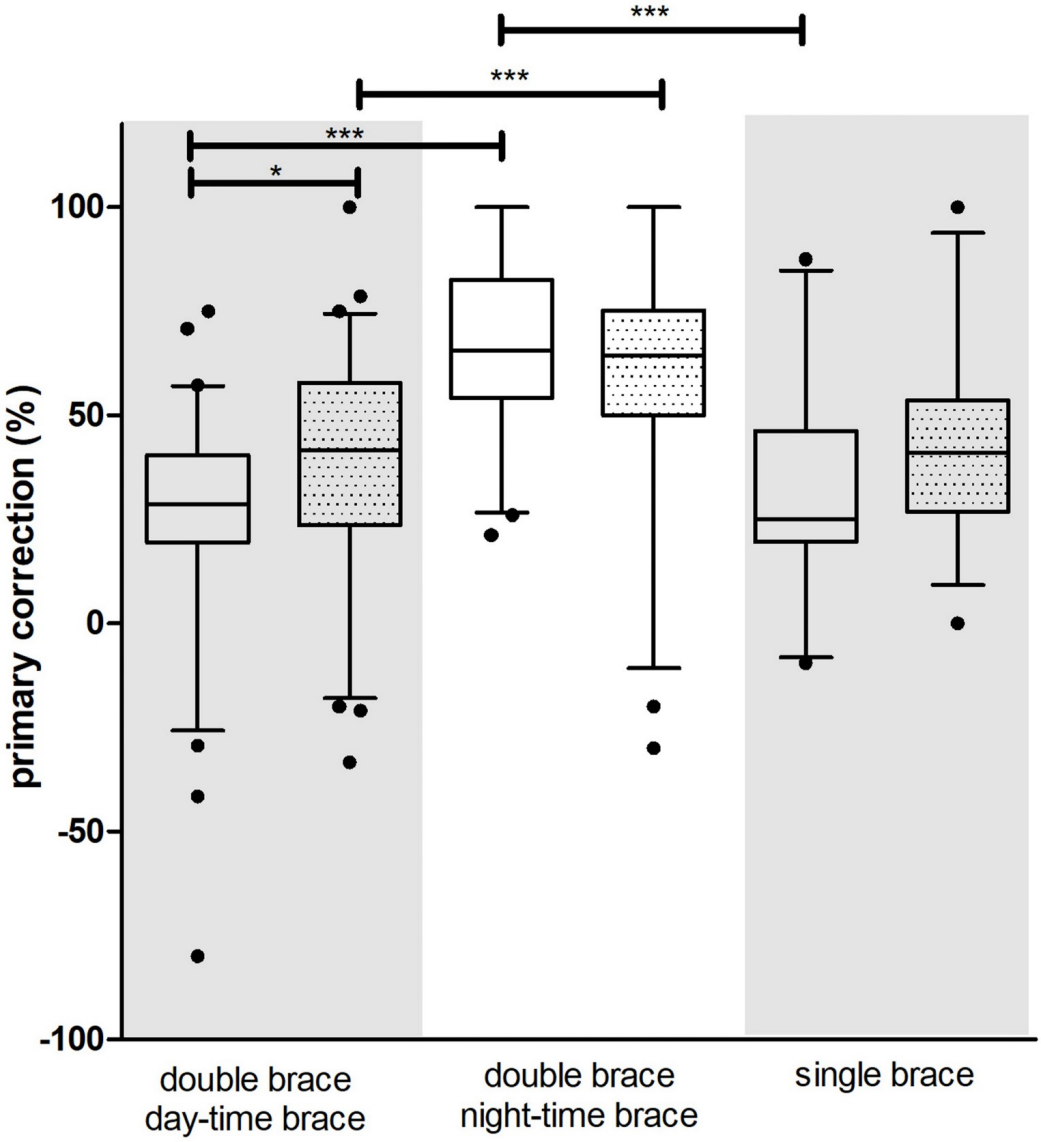
Primary correction (%) of brace therapy; plain boxplots thoracic spine values, dotted boxplots lumbar spine values, *** p< 0.001, * p<0.05, descriptive statistics can be found in the S1 Table.

The nighttime brace yielded a significantly higher primary correction than the daytime and single-brace therapy for both the thoracic and lumbar spine (Fig 4). Thereby the nighttime brace corrected an average of 18° ± 7° thoracic and 14° ± 7° lumbar (mean ± SD) (Table 1). There was a trend towards a higher primary lumbar correction with Chêneau type braces. (Table 1). Primary correction corresponds to the Cobb curvature angle with the brace tightened (angle, Table 1 line 2) and or the angle improvement to the initial value without the brace (percentage, Table 1 line 3). Effective correction means the angular degree of improvement (Table 1 line 4).

**Table 1:** Descriptive statistics of initial angle in degree Cobb angle and primary correction (mean ± SD), initial and follow-up angle in double brace, nighttime group equal to daytime.

	double brace, daytime brace thoracic	double brace, daytime brace lumbar	double brace, nighttime brace thoracic	double brace, nighttime brace lumbar	single brace thoracic	single brace lumbar
initial angle	27 ± 7	25 ± 7	-	-	26 ± 6	27 ± 7
primary correction (angle)	19 ± 6	15 ± 6	9 ± 6	10 ± 5	17 ± 7	14 ± 7
primary correction (%)	26 ± 25	39 ± 25	67 ± 21	60 ± 28	32 ± 21	44 ± 22
effective correction (angle)	7 ± 6	10 ± 7	18 ± 7	14 ± 7	10 ± 12	13 ± 11
follow-up angle	24 ± 9	19 ± 9	-	-	26 ± 9	22 ± 8

### 3.3. Cobb angle reduction

**Fig 5:**
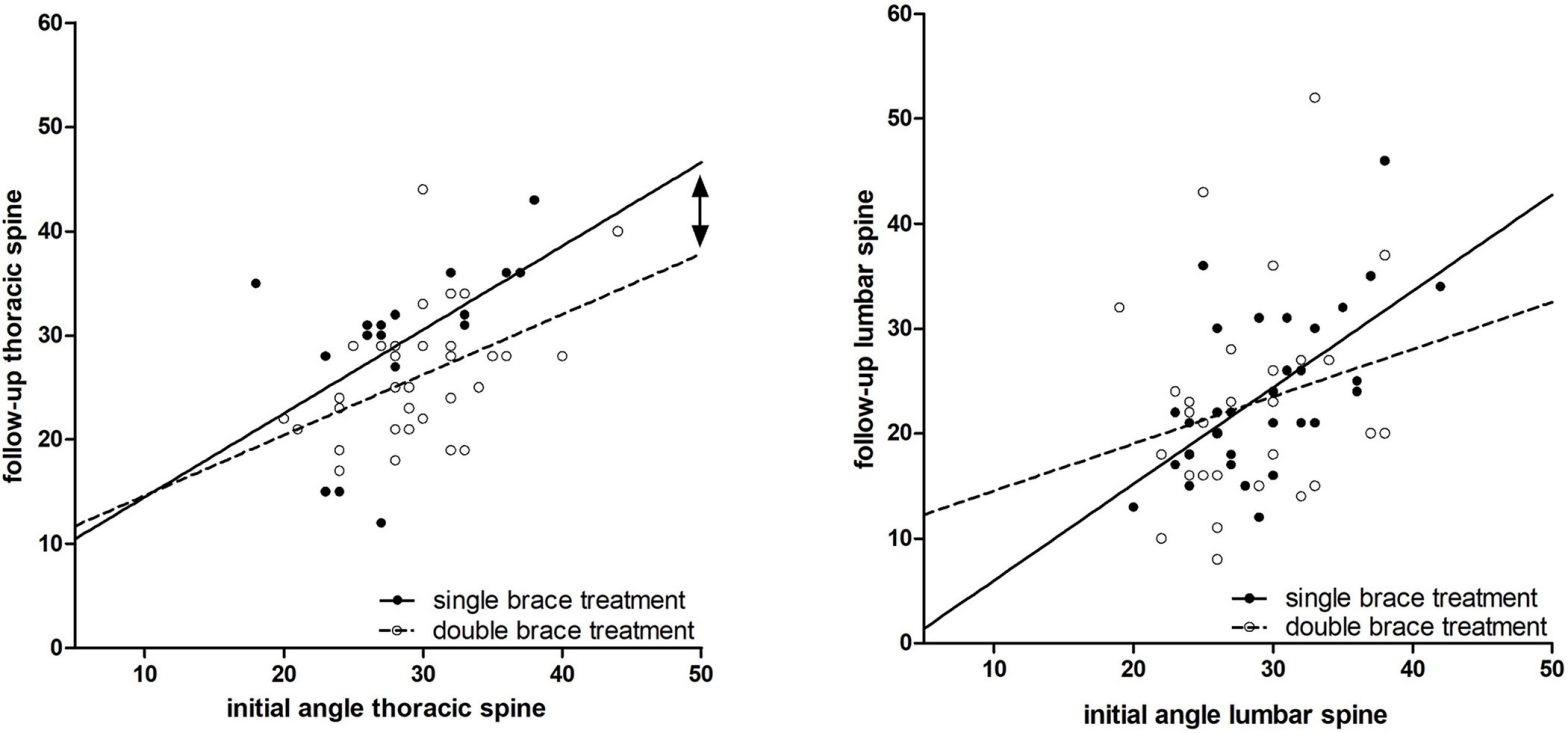
Follow-up Cobb angle (degree) in relation to the initial Cobb angle of the thoracic (left) and lumbar (right) spine, left: initial angle thoracic spine 29 ± 4, follow-up single brace 29 ± 8, double brace 25.94 ± 5.98, right: initial angle lumbar spine 29 ± 5, follow-up single brace 24 ± 8, double-brace 23 ± 10 (mean ± standard deviation).

Regression analysis of mean angles revealed an additional effect of about 5° improvement of double-braced thoracic curves, compared with single-brace therapy, where numbers showed a steady state (Fig 5). This effect was not visible in the lumbar area, where both groups showed an improvement of around 5°. Double-braced patients with a primary thoracic curvature showed a trend towards a lower angle in the follow-up examination after treatment compared to single-day therapy. We observed that a greater initial angle led to greater differences between the two treatment forms (Fig 5, arrow).

### 3.4. Treatment results

**Fig 6:**
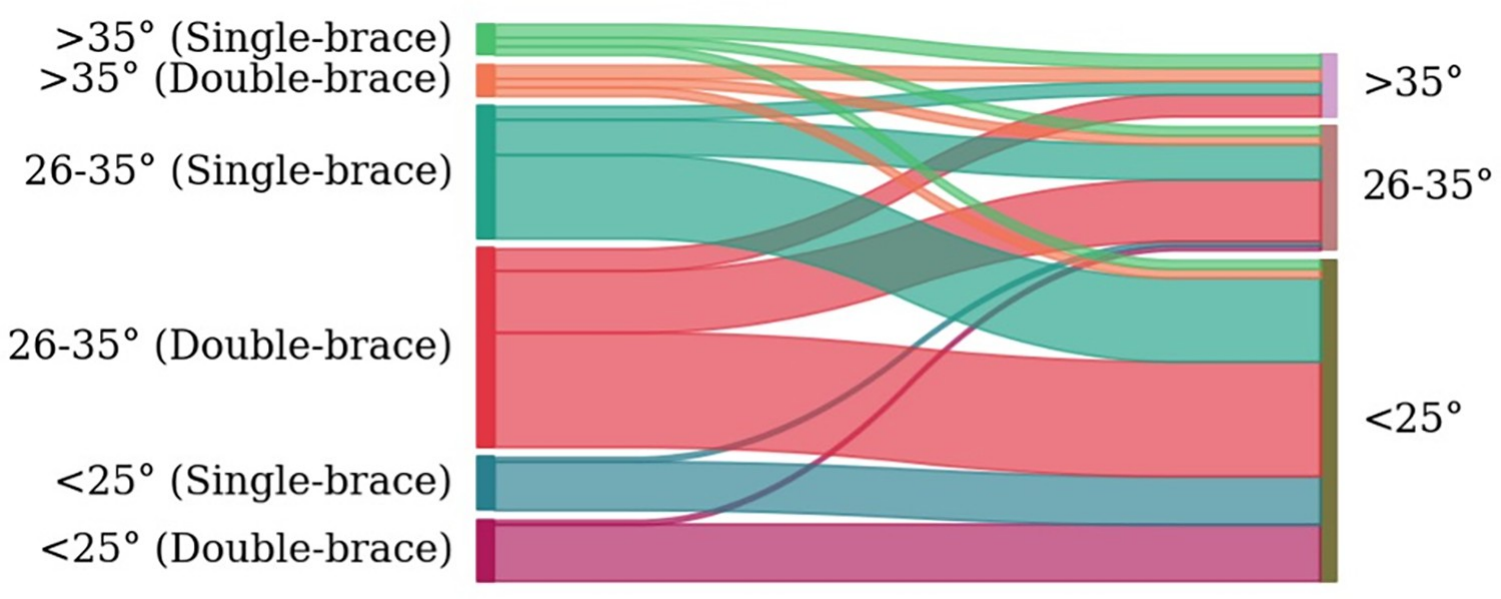
Distribution of treatment results in both treatment groups (red color schemes for double-brace group, green-color schemes for single-brace group) sorted categorial by primary initial COBB-angle value (left axis) and finale COBB-angle 2 years after brace weaning (right axis).

Both groups showed successful therapy in over 85% of cases. Of these, the majority of patients in both groups showed not only a steady state, but an improvement in Cobb curvature angle (Fig 6 and Table 2, p = 0.53). Single-brace therapy also leads to improvement in the final Cobb angle group in about 2/3 of the patients. Therefore, a final Cobb angle of less than 25°could be achieved in the majority of patients (Fig 6). The rate of insufficient therapy was lower in the double-brace group (10.6% vs. 14.3%, p = 0.58) (Table 2). Two patients from the double-brace group were operated (3%). There were no operations in the single-brace group, but one patient had a final Cobb angle above 45° (2%).

**Table 2:** Patient outcome 2 years after brace deposition (sufficient therapy ≤ 5°curve progression).

	Single-brace	Double-brace
**Sufficient therapy**	42 (85.7%)	59 (89.4%)
improvement (<0° progression)	25 (51.0%)	39 (59.1%)
steady state (0–5° progression)	17 (34.7%)	20 (30.3%)
**Insufficient therapy**	7 (14.3%)	7 (10.6%)

## 4. Discussion

We were able to show a success rate of 85% (single-brace) to 89% (double-brace) in a compliant brace-treated patient population with a follow-up of more than 2 years after maturity. Those values are in line with previous works and even superior to most other studies (Table 3).

**Table 3:** Comparison of success rate, case number, initial angle and follow-up period according to selected literature review (— = not reported).

Reference	Success	Failure = Surgery	Case-number	Mean Primary correction	Initial angle (range)	Follow-up
Nachemson et al. 1995 [19]	74% brace vs. 34% observation	--	111	--	25–36°	7–10 years
Danielson et al. 2001 [20]	84%	--	109	--	12–60°	22 years
Katz et al. 2001 [21]	61%	31%	51	25%	36–45°	32 months
	(33%-83%, depending on brace wearing time)					
Landauer et al. 2003 [22]	78%	0%	62	40%	20–40°	6 months
Bullmann et al. 2004 [23]	58%	27%	52	43%	25–40°	36–78 months
Vijvermans et al. 2004 [3]	74%	13%	151	--	25–40°	28 months
Seifert et al. 2009 [24]	89%	7%	96	30%	20–40°	24–84 months
Weinstein et al. 2013 [25]	72% braces vs. 48% observation	--	242	--	20–52°	24 months

Despite the SRS’s recommendation to include patients by intention to treat, we opted to exclude any patient with questionable compliance [2]. Non-compliant patients would have diluted the real difference between treatment groups. It is known from the literature that worse compliance also leads to lower correction results [6, 21].

The slight difference in scoliosis topology with a higher number of lumbar cases in the single brace group seems to be negligible. Any higher primary correction of lumbar curves does not materialize into better results at the end of treatment–as could be shown by our numbers and the work of Zaina et al. [26].

There was a higher primary correction in nighttime bracing compared to day-time braces. As no supine x-rays without brace were taken due to radiation exposure, we were not able to compute the brace effect from our cohort. But as the value of correction was 18° (mean, thoracic, Table 1 and 14° (mean, lumbar, Table 1) we observed a nighttime brace mitigated correction that was greater than the curve-flattening effect of supine positioning (10°) known from the literature [27]. Additionally, we could demonstrate that follow-up angles in double-brace treated patients tended to be lower than in single-braced individuals (Fig 5, Table 1). An additional nighttime brace was able to exert an additional durable correction force of about 5° in thoracic curves.

There was some evidence, that only additional nighttime bracing was able to permanently reduce thoracic Cobb angles in a few cases with progressed curves above 35° (Table 4). This effect can be attributed to the higher corrective forces relayed through the bending moment which is a result of asymmetric shoulder elevation. Whereas Cheneau-type braces are limited by scapular coverage which blocks effective derotation force application in cases where apical vertebrae of the thoracic curve are rather proximal, bending braces are able to squeeze out some more correction in proximal curves by the above-mentioned technique [12–14]. An additional factor is the flexibility of the deformed spine, which is specific to each patient whereby the primary correction result is influenced. Increased pressure point problems or insensitivity of the arm were not observed as a consequence.

However, speaking in clinical results we failed to demonstrate an overall supremacy of double-brace treatment in our treatment population. Compared to other studies our study comprises of rather large and homogeneous patient cohorts. Treatment results are in the expected range.

**Table 4:** Distribution of improvements in both treatment groups sorted by primary curvature and initial COBB-angle value (% of all treated patients with the respective brace therapy).

initial Cobb angle	<26°	26–35°	>35°
**Single-brace**			
T-Spine	2 (4.1%)	5 (10.2%)	-
L-Spine	4 (8.2%)	11 (22.4%)	3 (6.1%)
**Double-brace**			
T-Spine	2 (3.0%)	15 (22.7%)	3 (4.5%)
L-Spine	4 (6.1%)	12 (18.2%)	3 (4.5%)

This renders the number-needed-to-treat into ranges where cost-effectiveness is far out of reach. Statistical analysis revealed that we would have needed 530 patients in total to reach significance in comparing both treatments. This statistically significant difference of 5%, however, would have no clinical relevance in our opinion. Instead, the case number of 115 would have been sufficient to demonstrate a clinically relevant difference of more than 10 percent between the groups. As we did exclude non-compliant patients to observe a rather clean brace-methodological effect, we expect the number-needed-to-treat in a realistic setting to be even higher. This renders the supposed clinical effect of a customized add-on nighttime brace far too low to be cost-effective. Socioeconomic costs are more than doubled due to the add-on nighttime bracing with more complex fitting and the associated required extra x-rays.

From our data we can draw the conclusion that despite significantly higher primary correction, the customized add-on nighttime bracing does not provide a significant advantage in the 2-years follow-up.

Our study is limited by a slight intergroup asymmetry of regional angle distribution, which is not significant, but could have an effect in limit observation of treatment differences while analyzing overall good results. As another limitation, the time for which the brace was worn each day was reported by the patients or their parents, rather than being measured by means of a monitoring system [28]. The regular compliance survey was conducted at short intervals. The patients’ height and weight were not taken into account, although both of these factors could have affected the outcome of treatment.

There was also an age difference between the two groups. However, the single-brace group was on average 1 year older at the start of brace-therapy, which could have worsened their outcome. Nevertheless, the single-brace therapy was as effective as the double-brace therapy.

A possible bias could be due to the lack of randomization possibility because of the decision in favor of single-brace therapy by the health insurance company. Almost all patients in both groups had public health insurance, meaning there were no socio-economic differences between the groups in terms of insurance status.

In conclusion, add-on nighttime bracing fails to improve scoliosis brace treatment results at a population-wide scope in the 2-years follow-up.

## Supporting information

S1 Table In addition to Fig 4, the descriptive statistics of the primary correction in percent from initial Cobb angle can be found in the supporting information as S1 Table. (DOCX)
