# Supplementary material for: Impact of customized add-on nighttime bracing in full-time brace treatment of adolescent idiopathic scoliosis
Source: PLoS One. 2023 Jan 26;18(1):e0278421. doi: 10.1371/journal.pone.0278421 (PMC9879394; doi:10.1371/journal.pone.0278421)
Supplement: S1 Table — (DOCX) [file pone.0278421.s001.docx]

Table S1: Descriptive statistics of primary correction in percent from initial Cobb angle (Fig. 4)

|  | double brace, day-time brace  thoracic | double brace, day-time brace  lumbar | double brace, nighttime brace  thoracic | double brace, nighttime brace  lumbar | single brace  thoracic | single brace  lumbar |
| --- | --- | --- | --- | --- | --- | --- |
| 25% percentile | 19.38 | 23.67 | 54.17 | 50.00 | 19.62 | 26.79 |
| median | 28.57 | 41.67 | 65.63 | 64.50 | 25.00 | 40.97 |
| 75% percentile | 40.42 | 57.79 | 82.76 | 75.23 | 46.29 | 53.71 |
| mean | 26.19 | 39.22 | 66.59 | 59.88 | 31.57 | 44.10 |
| standard deviation (SD) | 24.93 | 25.17 | 21.41 | 28.11 | 21.45 | 22.45 |
| Lower 95% CI | 19.86 | 32.78 | 60.80 | 52.20 | 21.81 | 35.71 |
| Upper 95% CI | 32.52 | 45.67 | 72.38 | 67.55 | 41.33 | 52.48 |
